# Supplementary material for: Registered report protocol: Stress testing predictive models of ideological prejudice
Source: PLoS One. 2024 Aug 29;19(8):e0308397. doi: 10.1371/journal.pone.0308397 (PMC11361595; doi:10.1371/journal.pone.0308397)
Supplement: Attachment — Submitted filename: Response to Reviewer.docx [file pone.0308397.s002.docx]

We thank the editor and reviewer for their time, consideration, and valuable comments when reviewing our work. Below are our comments regarding the current incarnation of the paper. In this letter we include a combination of previous comments from the reviewer as well as those from the current round. Text in italics are our responses to specific points. Underlined italicized text are our responses, and non-underlined italicized text are quotes from the paper.

**Responses and Follow-up with the Reviewer From the Original Paper:**

In the first round of feedback we received, we addressed the reviewer’s concerns based on the PLOS One criteria survey completed by the reviewer and extensively detailed the changes that we made in a detailed response letter. We also pointed to the specific changes we made in the manuscript, included with tracked changes. We have re-included our previous comments alongside the response to the current reviewer comments to demonstrate how they were addressed when necessary, as well as added new comments and questions for the reviewer based on the current reviews.

**Journal Criteria:** 1. Does the manuscript provide a valid rationale for the proposed study, with clearly identified and justified research questions?

The research question outlined is expected to address a valid academic problem or topic and contribute to the base of knowledge in the field.

Reviewer #1: Partly

***Current Response:*** *Our original response to this is below, and it is quite detailed. We feel that we have sufficiently addressed this point/rating. However, there is no specific information in the review that we could use to know what the reviewer wants. We have put a lot of effort into making this work clear based on the first round of feedback. If the reviewer wants additional, specific changes for clarification of this point, please let us know what those specific changes are. We feel the changes we have made to the manuscript make the research question and its justification very clear.*

***Previous Response:*** *We thank the reviewer for this comment. As we edited the paper, we have made efforts to clarify our rationale for the proposed study. See pages 4 and pages 7-8 for two key examples of these changes:*

*(from page 4) “The prejudice and ideology literatures do not offer many concrete methodological suggestions for predicting the magnitude and direction of the effect of ideology on prejudice against specific groups. As an example, widely cited dual process models of prejudice suggest that a desire for social conformity and belief in a dangerous world predict right-wing authoritarian (RWA) attitudes, whereas the belief the world is a competitive jungle predicts social dominance, both of which predict prejudice [14]. Despite providing a theoretical rationale for the relationship between manifestations of ideology (i.e., RWA) and prejudice towards certain groups, this perspective does not provide scaffolding to predict the specific level of prejudice a person will exhibit towards a variety of target groups, in addition to examining a somewhat limited set of targets. This is a gap that should be filled because there is both practical and theoretical value in models that can predict the ideology-prejudice association across targets [4].”*

*(from pages 7-8) “The purpose of this project is to stress-test Brandt’s [4] original models using alternative measures of prejudice, including alternative explicit measures (i.e., relative measures of explicit prejudice), and reaction time measures (i.e., Implicit Association Test scores [20]). This addresses a key shortcoming of the original study. In particular, the original study used only feeling thermometers as an explicit measure of prejudice. Although feeling thermometers are common measures of prejudice (e.g., [10], [36], [18]), they are just one possible measure of group-based attitudes. The predictive accuracy of the models might be limited to feeling thermometers. If so, this would limit the utility of the predictive models to only studies that use feeling thermometers. This is a potentially substantial limitation given the diversity of prejudice measures that exist. However, if the predictive models work well with alternative measures of prejudice, i.e., the explicit and reaction time measures, it suggests that the model is much more generalizable. This would mean that this model could be used for different types of prejudice measures.”*

***Below we respond to criteria 2 and 3 simultaneously.***

**Journal Criteria:** 2. Is the protocol technically sound and planned in a manner that will lead to a meaningful outcome and allow testing the stated hypotheses?

Reviewer #1: Partly

**Journal Criteria:** 3. Is the methodology feasible and described in sufficient detail to allow the work to be replicable?

Reviewer #1: No

***Current Response:*** *We made extensive changes to the manuscript based on the reviewer’s comments regarding our initial submission. These changes were documented with tracked changes in the re-submission. The edits we made included such changes as further explaining how missing data will be handled up to current standards, adding additional clarification about how we will compute MSEs, and highlighted the advantage of our methods. In addition, we provide the full code we will use to analyze the data on OSF, which is linked in the manuscript on page (*<https://osf.io/buwp7/?view_only=3905d5d1d54b4a499483c03a089b9f6e>) *so anybody can see exactly which analyses we will run and how. This aids with clarity and replicability. The Ideology 2.0 dataset is a professionally collected, gold-standard dataset. It seems highly unlikely results from these data will not be replicable. Below we also include a few examples of our previous response addressing issues regarding methodological clarification and replicability, the full responses to all points are in our initial rebuttal letter. Without additional specific information from the reviewer however, we do not know what the reviewer wants. If the reviewer would like additional, specific changes we request they clearly state exactly what they want. We feel we have already addressed all comments given by the reviewer at time 1 regarding the method and replicability, and that both are clear.*

***Examples Of Our Previous Response to This Comment:*** *Additionally, we thank the reviewer for the request for clarification regarding post hoc tests and statistical software. We have added a clearer description of our strategy for our post hoc tests on page 21 (see quote below). For clarity, all analyses will be run using R Studio.*

*“Then, we will run a mixed ANOVA and post-hoc tests using the jmv package in R [30] to determine whether the differences between model MSEs are statistically significant (using an alpha level of .05). The model that has the lowest MSE (and the difference between that model and the next-lowest model is statistically significant) can be considered the most accurate predictive model of prejudice in this context. The calculations for the MSEs as well as the mixed ANOVA with post-hoc tests are included in the code on the project’s OSF page. The OSF Wiki contains details related to which lines of the code correspond to each calculation and test.”*

*Furthermore, we have added specific details about how MSEs are calculated on page 21 for the absolute measures (see quote below):*

*“We will estimate the mean squared error (MSE) of the observed ideology-prejudice association compared to the predicted association. MSEs serve as a means of evaluating the accuracy of a predictive model by comparing predicted and actual values. To calculate the MSEs, we will take the actual ratings of prejudice across targets, subtract them from the estimates from the equations, square these values, and then take the average.”*

*The MSE calculations for the relative measures are described on page 31 (see quote below):*

*“After we have trained our models using the training data, we will use the test data (the remaining 20% of the Ideology 2.0 dataset, for each target group) to estimate the observed relationship between ideology and comparative prejudice for each target group pair and see which predictive model captures observed comparative prejudice most closely. We will do this by estimating the MSE of the observed ideology-comparative prejudice association compared to the predicted association. By comparing the MSEs of each of the five models predicting the participant ideology-prejudice relationship across targets and the observed values of prejudice against each target in the test set, we can test which model(s) are most effective in predicting prejudice across targets. Lower MSEs when comparing the slopes in the estimates to the observed levels of participant ideology-comparative prejudice indicate better fit in this context.”*

***Below are the new comments from the reviewer, from the second round of feedback.***

Reviewer #1:

**Point 1** - Methodolgy is tough to interpret. How can data be accessed as it is gathered in stages.

*This is a registered report format* [*https://genweb.plos.org/RR/EditorResources_ONERegisteredReports.pdf*](https://genweb.plos.org/RR/EditorResources_ONERegisteredReports.pdf)*. In a registered report, the authors propose a study and method as well as detail the analysis to be conducted. After review, the paper may be accepted in-principle. This means that the journal agrees to publish the results, whatever they may be, if the authors undertake the proposed analyses in the agreed upon way. After in-principle acceptance the data are obtained, analyzed, and the manuscript is completed before being sent for review once more. In the paper, we describe on page 9 how the Ideology 2.0 data will be obtained. We are in contact with Schmidt and colleagues and they have agreed to provide us with the data upon stage 1 (in-principle) acceptance*

*“Here we propose testing four key models from Brandt [4], in addition to a null model (see Table 1) using a large dataset. Schmidt and colleagues [31] have issued a call for registered reports and have agreed to provide us with a new and very large (N = 261,119) dataset to conduct our analyses upon in-principle acceptance of a Stage 1 registered report. Studies 1 and 2 will use the Ideology 2.0 dataset [31].”*

*Furthermore, we describe how the data will be posted on OSF once it is obtained, and how it will be obtained on page 14.*

*“Across studies, we report how we determined sample size, how all participant exclusions were determined, and all manipulations and measures. All data used to derive this registered report and annotated R code (including proof-of-concept MSE estimates, which are described in the project OSF Wiki) are on the project’s OSF page:* [*https://osf.io/buwp7/?view_only=3905d5d1d54b4a499483c03a089b9f6e*](https://osf.io/buwp7/?view_only=3905d5d1d54b4a499483c03a089b9f6e)*. Upon acceptance of the Stage 1 registered report, we will pre-register the studies and planned analyses and then we will be given the full data. The full data will be posted on the project’s OSF page upon the final submission.”*

**Point 2** -Each data analytic step has not been meaningul explained e.g post-hoc tests were used by Turkey or Bonferroni?

*We attempted to clarify this in the first revision by stating that we would use an alpha of .05 (not an adjusted p value). There are any number of procedures one might use when making post-hoc comparisons and Tukey and Bonferroni are but a few. We do not wish to use these strategies however, because we do not wish to omit potentially meaningful effects (i.e., we are more concerned about type 2 errors than type 1 errors). We have further clarified this on page 23 of the manuscript.*

*“We will opt to use the original p values for the post-hoc tests as opposed to a procedure like Bonferroni or Tukey because we do not wish to omit potentially meaningful significant results based on stringent adjustments (Garcia-Perez, 2023).”*

**Point 3** -If data is not yet full or reached limit then must be waited for the complete findings.

*Our response to this is above. We cannot obtain or analyze the full data yet, as this is a registered report. To do so would violate the guidelines of a registered report* [*https://genweb.plos.org/RR/EditorResources_ONERegisteredReports.pdf*](https://genweb.plos.org/RR/EditorResources_ONERegisteredReports.pdf)*. What we have done is detail all steps that will be used in the analysis of the data when we obtain it using a very small subset of the data made available to us for the purposes of writing this stage 1 registered report as pilot analyses are allowed in PLOS One’s guidelines. If the paper is accepted in principle, we can obtain the data and complete the work, but we cannot do so until we are at that stage.*
